# Harnessing Artificial Intelligence for Intravascular Imaging: Is it Percutaneous Coronary Intervention Ready?∗

**DOI:** 10.1016/j.jacadv.2023.100565

**Published:** 2023-08-22

**Authors:** Partho P. Sengupta, Chirag Bavishi

**Affiliations:** a Division of Cardiovascular Diseases and Hypertension, Robert Wood Johnson University Hospital, and Rutgers Robert Wood Johnson Medical School, New Brunswick, New Jersey, USA; b Division of Cardiovascular Medicine, University of Missouri at Columbia and MU School of Medicine, Columbia, Missouri, USA

**Keywords:** artificial intelligence, IVUS, machine learning, PCI

**Figure:**
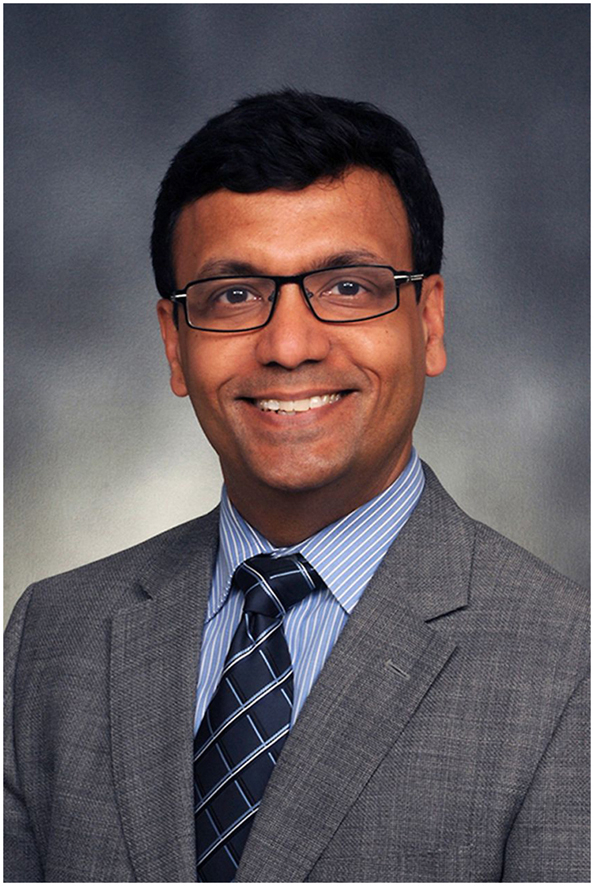

Since its introduction in the 1980s, intravascular ultrasound (IVUS) has significantly contributed to the quantitative analysis of coronary artery stenotic lesions and the advancement of percutaneous coronary interventions (PCIs). Despite its crucial role, however, the global adoption of preintervention IVUS is limited due to the procedural complexities that require added time and substantial expertise necessary for optimal image interpretation. The advent of artificial intelligence (AI) may overcome challenges in the IVUS interpretation process through innovative techniques for image processing, feature extraction, plaque identification, and automated quantitation.

A prime component of AI is machine learning (ML), a repertoire of techniques facilitating AI to learn and evolve. Within the ML spectrum lies deep learning, a paradigm mimicking the intricate networks of the human brain, now being applied increasingly in cardiovascular imaging to enhance automation and precision. In this issue of *JACC: Advances*, the study led by Matsumura et al^1^ examined the accuracy of deep learning in automating the segmentation of coronary artery vessel and lumen dimensions, including balloon sizing, using high-definition 60 MHz IVUS images. The team employed the U-Net convolutional neural network (CNN) algorithm, training their model against the gold standard of expert analysis. A total of 8,076 IVUS images formed the backbone of the model's training and validation, while a secondary set of 437 IVUS images served as the test bed for independent evaluation. The ML model exhibited high alignment with expert segmentation in the primary data set, posting correlation coefficients of 0.992 and 0.993 for lumen and vessel areas, respectively. This trend remained consistent in the independent data set, with correlation coefficients of 0.991 and 0.967 for lumen and vessel areas, respectively.

The primary end point analyzed the congruence of balloon size selection between the ML model and expert analysis, yielding agreement rates of 70.6% (based on vessel diameter alone) and 92.4% (by including lumen diameter) in the independent data set. Notably, agreement rates rose when vessel borders were visible, indicating that visibility significantly influenced balloon sizing errors. Using a lumen area difference of <0.5 mm^2^, the agreement rate stood at 85.5%, while this rate surged to 97% for the acute stent area. The levels of agreement and mean differences for both lumen and vessel areas remained similar between the ML model and the expert and 2 interventional cardiologists.

CNNs offer superior performance in analyzing visual data, utilizing a hierarchical approach to efficiently learn complex patterns, reducing reliance on hand engineering, and achieving memory efficiency through weight sharing.^2^ The present study used a CNN-based architecture to enhance the interpretation of IVUS images and help choose balloon sizing during IVUS-guided PCIs. Although the performance of CNN-based segmentation was good, there were shortcomings in performance level largely due to suboptimal images obscuring vessel visibility. For any AI technique, the quality and scope of the data determine the applicability and accuracy of the algorithm, regardless of the approach. Pertaining specifically to IVUS images, several variables, such as the vessel wall architecture, calcification, plaque burden, aneurysms or ectasia, and dissection, can affect image quality and, consequently, its interpretation. These variables also impact human understanding. However, for human operators, in this case, interventional cardiologists, their clinical training, knowledge base, experience, and expertise can help surmount these challenges, an area where current AI platforms fall short. In the present study, the balloon size selection agreement achieved a high of 95.3% even when the vessel borders were well visualized. Balloon dilatation post-PCI is an essential procedural step to optimize the stent; however, catastrophic complications such as coronary artery perforation may occur in incorrect balloon size selection. In a 16-year study of 24,465 patients undergoing PCI at 2 medical centers in Italy, it was observed that in 50% of cases of Ellis Grade III coronary perforation, the device causing the perforation was an intracoronary balloon.^3^ The perforation occurred during predilation before stent implantation in 39.3% and during postdilation following stent deployment in 60.7% of the patients. Considering the lack of 100% accuracy of AI in interpreting vessel characteristics, implementing automated AI as a decision tool during IVUS, at the present stage, will still require human-in-loop for oversight. Eventually, computer vision and cognitive computing advancements may overcome some of these limitations.^4^

The present study adds to the growing literature showing high level of agreement between deep learning-based models and manual expert analysis for IVUS interpretation during PCI. In a study by Shinohara et al,^5^ compared to manual segmentation, U-Net CNN-based algorithms showed a strong correlation of 0.97 for vessels with significantly narrowed lumen (<4 mm^2^) and 0.98 for those with severe calcification using high-definition 60 MHz IVUS images. However, in this study, the algorithm had difficulty in accurately identifying stents. In the study by Nishi et al,^6^ DeepLab3-based CNN algorithm showed a high agreement with expert analysis for lumen, vessel, and stent areas (correlation coefficients of 0.98, 0.96, and 0.96, respectively) using 40 to 45 MHz IVUS images. Deep learning algorithms have also been developed for evaluating plaque characterization, calcification, and stent characteristics from IVUS images.^7^^,^^8^

AI techniques hold immense promise. However, their adoption in busy interventional practice, particularly for imaging-based interventions such as IVUS, is beset with many challenges. The evolution of robust clinical evidence will require a standardization of image acquisition protocols and a dire necessity for randomized and pragmatic clinical trials—all underpinned by reliable multicenter clinical databases and federated learning designs. Moreover, implementing AI in the complex architectures of ever-evolving clinical environments is a herculean task, necessitating consistent updates and vigilant oversight for maintaining efficacy. The advent of large-scale architectures such as generative pretrained transformers open a new horizon for a democratized AI landscape.^9^ These models, which may soon process multimodal feeds like language, image, and video, could seamlessly integrate IVUS image-based models with existing patients’ electronic medical records and outcomes, enhancing procedure planning and interventional strategy selection. Nonetheless, the fidelity, ethical implications, and privacy concerns surrounding these architectures have instigated considerable debates.^10^ Despite these uncertainties, there is little doubt that health care is reaching a pivotal juncture, offering unprecedented opportunities for harmonious integration of imaging, intelligence, and automation for personalized interventions.

## Funding support and author disclosures

Dr Sengupta serves as an advisor for Echo IQ and RCE Technologies.
